# Case-control study of the efficacy of retrogastric Roux-en-Y choledochojejunostomy

**DOI:** 10.18632/oncotarget.16006

**Published:** 2017-03-08

**Authors:** Xin-Wei Yang, Jun-Yi Chen, Wen-Liang Yan, Jing Du, Zhi-Jian Wen, Xing-Zhou Yan, Ping-Hua Yang, Jue Yang, Bao-Hua Zhang

**Affiliations:** ^1^ Eastern Hepatobiliary Surgery Hospital, Second Military Medical University, Shanghai, China; ^2^ Department of General Surgery, the Fourth People's Hospital of Shanghai, Shanghai, China; ^3^ Department of Dermatology, Jinling Hospital, Nanjing, China; ^4^ Second Military Medical University, Shanghai, China

**Keywords:** delayed gastric emptying, cholangitis, choledochojejunostomy, obesity, prognosis

## Abstract

The traditional, retrocolic/antegastric Roux-en-Y choledochojejunostomy is technically complicated, and the incidence of postoperative complications remains high. Here we report the outcome of 59 consecutively treated patients (*study group*, SG) that underwent a new choledochojejunostomy method in which the jejunal loop is passed behind the antrum pyloricum (retrogastric route). A retrospective comparison was made between this group of patients and 187 patients (*control group*, CG) that underwent conventional Roux-en-Y choledochojejunostomy (antegastric route). Baseline clinicopathological characteristics were similar in both groups, except for the BMI, which was significantly higher in the SG. The time spent on constructing the anastomosis, as well as overall postoperative complications, did not differ between groups. Compared with the CG, the incidence of postoperative delayed gastric emptying was decreased in the SG, and the time elapsed before the patients’ first postoperative liquid food consumption was shorter. We ascribe these beneficial effects to the superiority of the modified, retropyloric choledochojejunostomy approach, and propose that this surgical technique is particularly suitable for obese patients, especially those with a short ascending bowel loop.

## INTRODUCTION

Biliary obstruction is associated with renal failure, body fluid disturbances, and myocardial dysfunction. Roux-en-Y choledochojejunostomy is a common surgical procedure for resolving extrahepatic biliary obstruction and reconstructing biliary-enteric continuity after resection for benign or malignant biliary diseases [1]. The conventional Roux-en-Y choledochojejunostomy is commonly performed using the retrocolic, antegastric route, and is associated with many complications, including bile leakage, reflux cholangitis, anastomotic strictures, delayed gastric emptying (DGE), and other postoperative complications [2–8]. Since these complications may be determined by this conventional route of biliary-enteric anastomosis, it is necessary to pay more attention to the phenomenon.

With ongoing experience surgeons are trying to find improved choledochojejunostomy procedures to avoid them. Although various surgical modifications have been proposed, the results remain controversial and postoperative and long-term complications are still common [5–8] [9–15].

DGE without mechanical obstruction can occur in the postoperative period after upper gastrointestinal tract surgery, such as after gastric surgery and choledochojejunostomy [16–18]. With advances in operative techniques, intensive care medicine, interventional radiology, and better patient selection and preparation, the perioperative mortality of pancreatic and biliary surgery in high-volume centers has decreased markedly over the past two decades [16, 19]. Despite this improvement in mortality, postoperative morbidity remains high, and DGE continues to be one of the most common postoperative complications after biliary surgery [16, 19, 20].

Considering all this, an objective reevaluation of choledochojejunostomy is meaningful. During our surgical experience, we developed a modification of the biliary-enteric anastomosis procedure using a route in which the jejunal loop passes behind the antrum pyloricum. In this report, we compared the outcomes of the new retropyloric biliary-enteric reconstruction procedure with that of the traditional antegastric Roux-en-Y choledochojejunostomy.

## MATERIALS AND METHODS

A prospectively maintained hepatobiliary surgery database was reviewed for all patients who underwent choledochojejunostomy in our department between January 2007 and December 2012. A total of 246 patients that received Roux-Y choledochojejunostomy were assigned to one of two groups: (1) Control group, CG (n = 187), where patients underwent retrocolic, antegastric reconstruction, and (2) Study group, SG (n = 59) where choledochojejunostomy was performed via the retropyloric (s.s. behind the antrum pyloricum) route. We compared retrospectively the clinical outcomes of the two groups, focusing on the manifestation of postoperative gastrointestinal disorders and the time elapsed before resuming liquid food consumption after surgery.

### Ethics statement

All studies were approved by the Committee on Ethics of the Second Military Medical University. Written consent was given by the patients for their information to be stored in the hospital database and used for research upon admission.

### Inclusion and exclusion criteria

The inclusion criteria were as follows: (1) Either a lack of residual biliary stones as determined by postoperative cholangiography or complete removal of residual stones by postoperative choledochoscopy; (2) complete removal of any congenital bile duct cystic dilation with no postoperative residual lesions; (3) a survival time of at least six months and no recurrence within this period (cancer patients); (4) complete follow-up data.

### Definitions

The occurrence of right upper abdominal pain, chills, fever, or jaundice within six months after discharge was defined as acute cholangitis attack. Chronic cholangitis was defined by the presence of these symptoms 6 or more months after surgery. Chronic cholangitis was caused either by bacterial invasion or by obstruction of the ducts by calculi or a tumor. The condition is characterized by severe right upper quadrant pain, jaundice (if an obstruction is present), and intermittent fever. Blood tests reveal an elevated level of serum bilirubin. Treatment consists of antibiotics for infection and surgery for acute obstruction. In patients with cholangitis, magnetic resonance cholangiopancreatography was used to assess whether the complication was caused by an anastomotic stricture or by reflux due to roux loop stasis. In our study, DGE was defined as any combination of the use of a nasogastric (NG) tube for 10 days or more, regular diet not tolerated by postoperative day 14, or the reinsertion of an NG tube, as suggested by Tani and coworkers [21].

### Demographic data

A total of 398 cases of cholangioenterostomy were recorded in our hospital between January 2007 and December 2012. According to the inclusion and exclusion criteria, 246 eligible patients were included in our study. 59 cases (27 males and 32 females; average age of 52.6 years) with modified, retropyloric Roux-en-Y cholangioenterostomy, were included in the study group (SG). Of these, 13 patients (22.0%) had a history of hepatobiliary surgery. 187 cases (64 males and 123 females; average age of 50.4 years) with conventional, antegastric Roux-en-Y cholangioenterostomy were included in the control group (CG). The two groups of patients showed good comparability regarding type of primary disease, age and gender distribution, previous history of biliary surgery, and preoperative liver function (Table 1).

**Table 1:** Clinicopathological characteristics of patients in the study group (SG) and control group (CG)

Variable Value	SG (n=59)	CG (n=187)	P Value
Age	52.6±14.1	50.4±14.8	0.604
Male gender	27	64	0.110
BMI	25.4	19.8	0.001
Hospital stay(days)	22.0±8.1	21.1±9.0	0.994
Primary disease			0.259
Hepatolithiasis	6	44	
Choledochal cyst	12	43	
Intrahepatic cholangiocarcinoma	7	9	
Gallbladder cancer	9	13	
Hilar cholangiocarcinoma	14	37	
Distal cholangiocarcinoma	6	13	
Reflux cholangitis	5	28	
Biliary operation history			0.239
Present	13 (22.0)	56 (29.9)	
Absent	46 (78.0)	131 (70.1)	
Combined liver resection	27 (45.8)	74 (39.6)	0.074
Segment or lobe resection	20	58	
Partial hepatectomy	7	16	
Plane of biliary-enteric anastomosis			0.468
Common hepatic duct	31	100	
Bifurcation of the common hepatic duct	12	56	
Left and/or right hepatic duct	16	31	
Anastomotic diameter	2.30±0.45	2.46±0.50	0.123
Operative time (min)	258.8±59.4	269.1±61.0	0.654
Biliary-enteric anastomoses	26.5±8.9	31.8±15.6	0.124
Enteroenterostomy	25.8±9.4	34.7±10.8	0.341
Blood loss	393.2±237.9	373.5±200.5	0.244

### Surgical procedures

**Figure 1:**
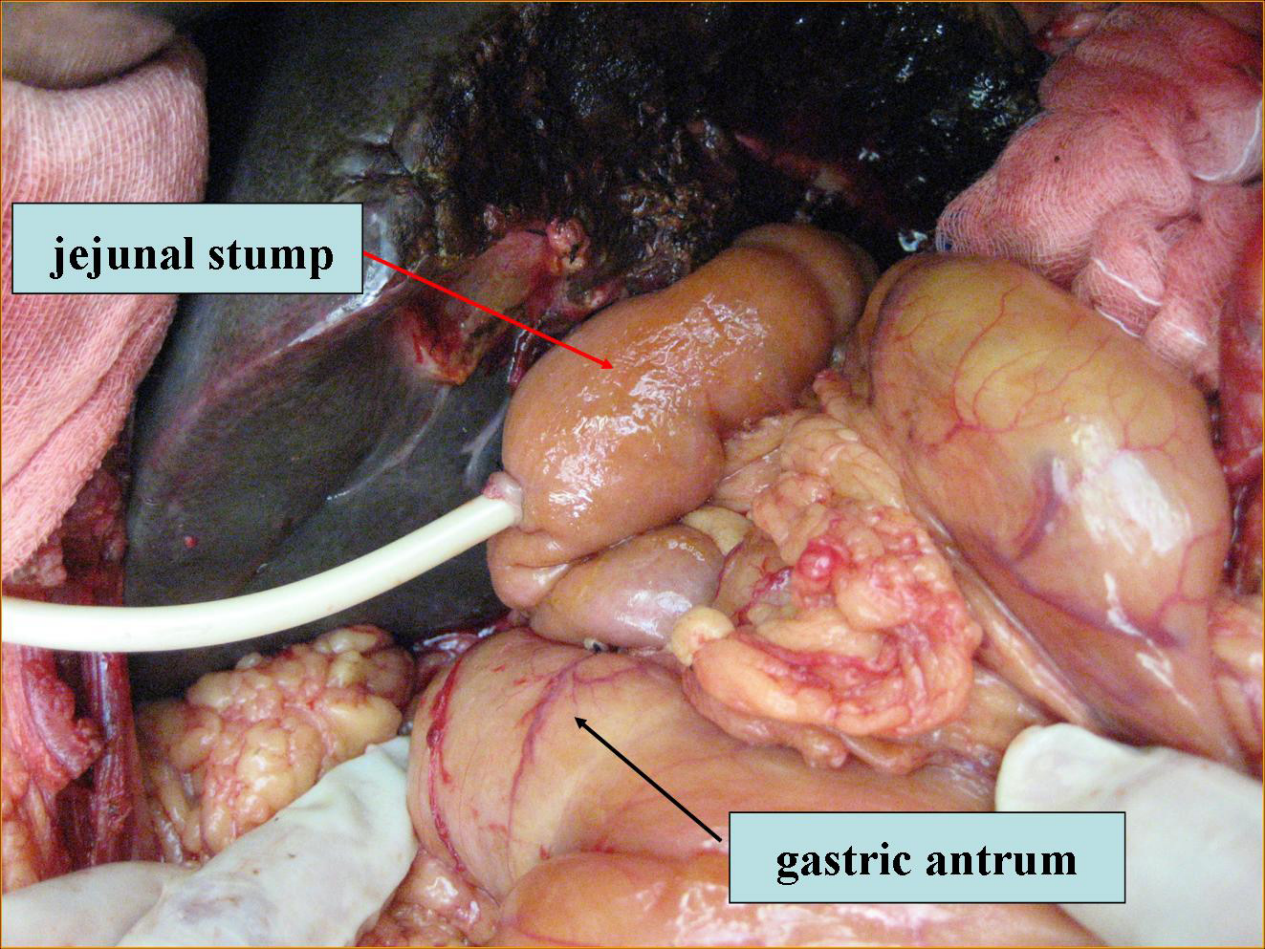
Retrogastric Roux-en-Y choledocho-jejunostomy

Modified, retropyloric choledochojejunostomy (study group; SG): After resection of the primary lesion, all 59 consecutive patients underwent biliary-enteric Roux-en-Y choledochojejunostomy using a retrogastric (s.s. retropyloric, i.e. passing behind the antrum pyloricum) route under general anesthesia (Figure 1). A T-tube was inserted within the biliary-enteric anastomosis whenever necessary, and left in place for 5 to 14 weeks. The distal jejunal stump was raised to the hepatic hilum behind both the transverse colon and the gastric antrum, then through the fascia between the stomach and pancreas, finally piercing through the superior mesenteric root. A 5-0 absorbable suture was used for interrupted suturing of the biliary-jejunal anastomosis.

**Figure 2:**
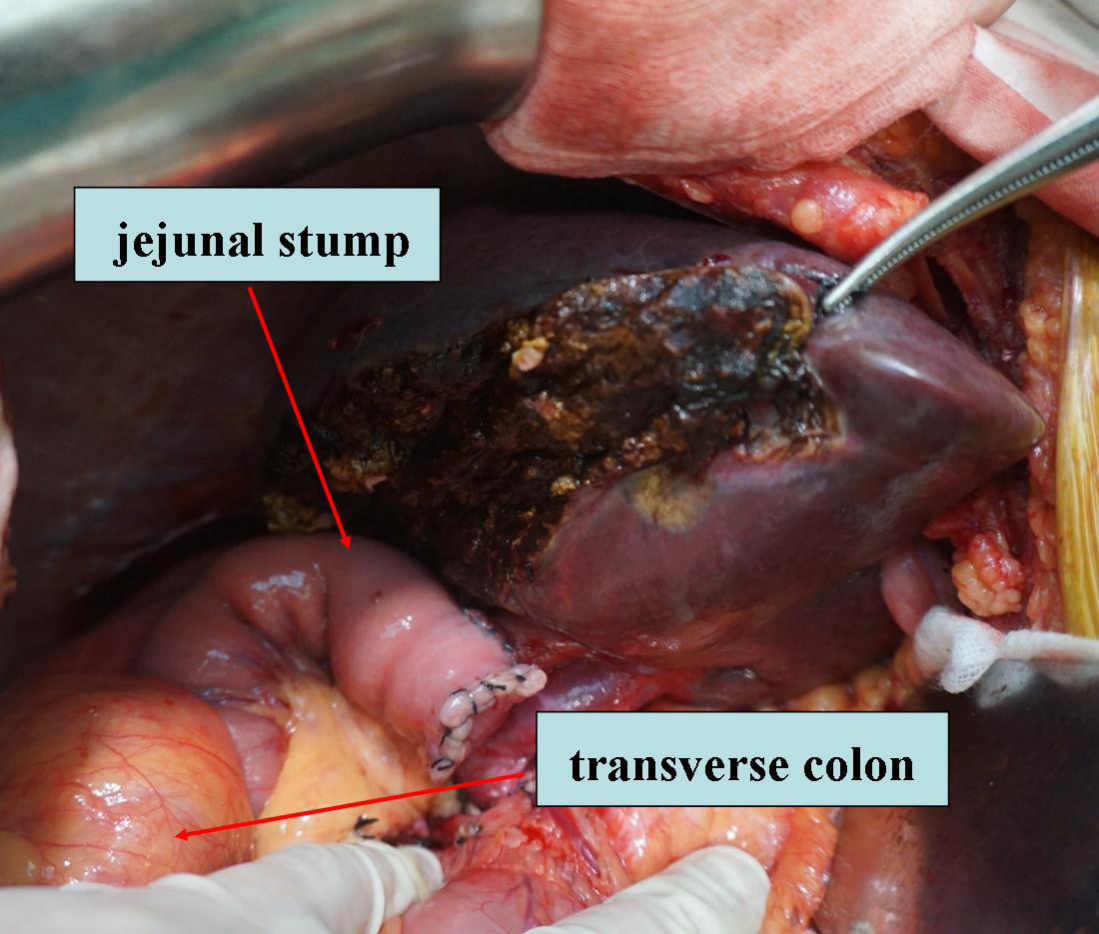
Retrocolic and antegastric Roux-en-Y choledochojejunostomy

Conventional, antegastric choledochojejunostomy (control group, CG): The surgical procedure was the same as in the modified anastomosis approach (SG) except that the biliary-enteric anastomosis was reconstructed using an antegastric (i.e. passing in front of the pyloric antrum) route (Figure 2).

### Perioperative outcome and follow-up

The follow-up was mainly completed by out-patient reviews and by telephone interviews. Patients’ symptoms were primarily registered during out-patient follow-up. The median follow-up time was 4.8 years (range: 36 months to 9 years). Clinicopathological data such as operation time, time elapsed until consumption of the first postoperative liquid meal, and incidence of postoperative acute or chronic cholangitis were compared between two groups. The incidence of acute cholangitis, bile leakage, and postoperative anastomotic strictures (within six months), as well as the time until ingestion of a liquid meal, and the incidence of postoperative DGE, were the most important evaluation indexes. However, because anastomotic strictures could be caused by recurrent tumors or anastomotic stomas, long-term anastomotic strictures were not further analyzed.

### Statistical analysis

Continuous data were expressed as mean ± SD or median (range). Variables were compared by the χ^2^ test, Fisher's exact test, or Mann-Whitney's U test, where appropriate. A P value <0.05 was considered to indicate statistical significance. Analyses were performed using the SPSS statistical package (version 11, SPSS, Inc, Chicago, IL).

## RESULTS

### Clinicopathological data and operative details

Patients that underwent conventional (retrocolic, antegastric) Roux-en-Y anastomosis (control group, CG) or modified (retrocolic, retrogastric; s.s. retropyloric) Roux-en-Y anastomosis (study group, SG) showed good comparability in regards to type of primary disease, age and gender distribution, previous history of biliary tract surgery, preoperative liver function and other general clinical aspects (Table 1). There was significantly higher BMI in the SG than CG (P=0.001). There was no statistical difference between the two groups in the extent of combined hepatic resection, operative time, biliary-enteric anastomotic plane, or anastomotic diameter (Table 1). The time required to complete the biliary-entero anastomoses (26.5 ± 8.9 min for the SG vs. 31.8 ± 15.6 min for the CG; P = 0.124) and entero-enteric anastomoses (25.8 ± 9.4 min for the SG vs. 34.7 ± 10.8 min for the CG; P = 0.341) was appreciably shorter in the SG group, but the differences did not reach statistical significance (Table 1).

### Comparison of postoperative efficacy

The incidence of overall postoperative complications did not differ between the two groups. There were also no differences between groups in the incidence of bile leakage. The incidence of postoperative DGE was, however, significantly decreased in the SG (P = 0.031), as was the time to first liquid meal ingestion (P = 0.004). Within six months after discharge, the incidence of acute cholangitis in the SG (2 patients; 3.4%) was lower than in the CG (14 cases; 7.5%). However, this difference was not statistically significant (Table 2). One patient with acute cholangitis attack in the SG group was cured by oral antibiotics. Of the 14 patients with acute cholangitis in the CG, 6 cases were relieved by oral anti-inflammatory analgesic, and 8 patients required hospitalization for further therapy. The ratio of patients requiring hospitalization for acute cholangitis was higher in the SG than in the CG, but this difference was not statistically significant (Table 2).

**Table 2:** Comparison of postoperative efficacy

Variable Value	SG (n=59)	CG (n=187)	P Value
Bile leakage	2 (3.4%)	11 (5.9%)	0.457
Fluid consumption (hours)	66.4±17.4	76.3±25.5	0.004
Delayed gastric emptying	1 (1.7%)	20 (10.7%)	0.031
Postoperative complications (need of invasive treatment)	6 (10.2%)	16 (8.6%)	0.706
Acute cholangitis	2 (3.4%)	14 (7.5%)	0.267
Required hospitalization	1 (1.7%)	8 (4.3%)	0.358
Chronic cholangitis	1 (1.7%)	10 (5.3%)	0.238
Required hospitalization	1 (1.7%)	9 (4.8%)	0.291
Anastomotic strictures	1 (1.7%)	7 (3.7%)	0.440

### Comparison of long-term prognoses

A total of eleven patients presented with postoperative chronic cholangitis. Only one belonged to the SG (1.7% incidence) while the other 10 cases (5.3% incidence) occurred in the CG. However, the incidence of chronic cholangitis, as well as the postoperative incidence of anastomotic strictures within six months after discharge, did not differ significantly between the two groups.

## DISCUSSION

Since the Roux-en-Y reconstruction technique was first reported in 1893, biliary-enteric anastomosis routes have been greatly improved [9]. However, the jejunal stump or “bridge loop” placement has typically remained antegastric. Since 2007, we began to suspect the rationality of this approach. Thus, we tested a new retrogastric route, i.e. traversing behind the antrum pyloricum, to raise the jejunal stump during biliary-enteric anastomoses and compared the clinical outcomes with those of patients receiving conventional, antegastric Roux-en-Y choledochojejunostomies.

Reflux cholangitis has been reported as the most common complication of Roux-en-Y hepatojejunostomy [10]. Reflux cholangitis may result in anastomotic strictures, stone recurrence, and liver abscesses [11]. Long-term reflux cholangitis could also increase the risk of tumorigenesis [12]. A variety of anti-reflux procedures and devices have emerged to solve this complication. Among these, the anti-reflux effects of artificial nipples have been inconsistent because of chronic fibrosis and gradual impairment of the countercurrent flow [22]. For example, when a silicone anti-reflux valve was placed between the bile duct and intestine to prevent reflux of the intestinal contents, the clinical effect was not sustained. In addition, anti-reflux devices can potentially increase the difficulty of surgery and impose a financial burden on patients [22, 23, 24]. In conventional Roux-en-Y choledochojejunostomies the proximal jejunal loop is usually long and has an inverted C shape. Contents in the proximal jejunum easily enter the distal jejunum upon anastomosis and then retrogradely reach the bile duct, a risk factor associated with postoperative reflux cholangitis. Based on these anatomical features, we changed the C-shaped curve present in the conventional, antegastric route into a straight line in our retrogastric (retropyloric) approach, preserving a more natural state of the bridge loop and its mesentery. The aim of this modification was to effectively avoid the risk of reflux cholangitis while meeting the requirement of a tension-free anastomosis.

### Decreased delayed gastric emptying after retrogastric choledochojejunostomy

With the advent of innovative techniques, including operative techniques, intensive care medicine, interventional radiology, and novel pharmacological agents, the mortality rate after choledochojejunostomy has greatly decreased. Despite this improvement in mortality, postoperative morbidity remains high, and much of this is due to DGE, which remains one of the most common complications after upper gastrointestinal tract surgery. Although for most patients DGE is not life-threatening, it can cause discomfort, increase the duration of postoperative hospitalization, increase hospital costs, and decrease quality of life. The mechanisms of postoperative gastroparesis, gastric stasis, and DGE are still poorly understood [16, 25]. Operative factors such as the method of gastric drainage reconstruction (antecolic versus retrocolic) may impact the incidence of DGE [26, 27]. Several reports suggested that other postoperative complications increase the incidence of DGE [16, 25, 27], which is often, but not always, associated with pancreatic fistula, peripancreatic collections, or intraabdominal abscesses.

Currently, with fast-track surgery and reduced length of hospitalization becoming increasingly common for patients undergoing hepatobiliary surgery, the direct association between DGE and prolongation of hospital stay has substantive economic impact. International differences regarding the duration of the postoperative hospital stay must be acknowledged when considering this topic, which has been addressed in a previous study carried out in our hospital [28].

Many risk factors associated with DGE have been reported after choledochojejunostomy. In Roux-en-Y biliary-enteric anastomosis the jejunum is usually transected, and its motility and electrical activity are greatly impacted. This can cause reverse peristalsis [29], and as a result, the intestinal contents can become stagnant, thus favoring bacterial colonization and leading to postoperative cholangitis and abnormal liver function [30]. Due to the weight of the transverse colon and the gastric antrum, the pylorus and duodenum can be compressed in choledochojejunostomies performed using the antegastric route. This is not conducive to the transmission of the gastrointestinal peristaltic rush and is associated with a greater risk of gastrointestinal disorders, especially when the patient is in a supine position. Intestinal obstruction caused by compression of the gastric pylorus or duodenum can be prevented by re-routing the procedure using the retrogastric (s.s. behind the pyloric antrum) approach. This is instead conducive to a better postoperative recovery of gastrointestinal function. As our results show, the incidence of postoperative DGE was significantly decreased in our study group.

### Retropyloric choledochojejunostomy is particularly suitable for obese patients

We propose that this new technique is particularly suitable for patients with obesity. Because a pachyntic pylorus is common in obese patients, creating a C-shaped jejunal curve located anteriorly to the pyloric antrum during biliary-enteric anastomosis is more difficult and more prone to reflux cholangitis. Obese patients are more likely to have relatively short mesenteries, especially patients with abnormal vascular arches, and the length of the ascending bowel loop is often shortened. Because the portal hilum is usually buried deep after hemihepatectomy, longer lengths of bowel and mesentery are required for biliary-enteric anastomosis using this conventional route. In this situation, the risk of bile leakage greatly increases because of increased tension. Re-routing the anastomosis behind the pyloric antrum can mitigate these risks, as evidenced in our previous report [31].

Our study presents some limitations, mainly regarding its retrospective design and the small number of patients studied. Further evaluation of larger numbers of patients, including prospective studies, are thus required to confirm our results.

In conclusion, a retrogastric approach during Roux-Y choledochojejunostomy decreases the incidence of DGE and reduces the time elapsed until consumption of the first postoperative liquid meal. Our data suggests that this modified anastomosis route is safe and efficacious, especially for obese people.

## Abbreviations

MAG: modified anastomosis group
CG: control group
CJ: choledochojejunostomy
ALT: alanine transaminase
AST: aspartate aminotransferase
ALP: alkaline phosphomonoesterase
G-GT: gamma-glutamyl transferase
